# Metastatic Gastric Signet Ring Cell Adenocarcinoma Presenting With Colonic Stenosis

**DOI:** 10.7759/cureus.8420

**Published:** 2020-06-03

**Authors:** Jurij Hanzel, Branislava Ranković, Marija Ribnikar

**Affiliations:** 1 Gastroenterology, University Medical Centre Ljubljana, Ljubljana, SVN; 2 Pathology, University of Ljubljana, Ljubljana, SVN

**Keywords:** metastasis, secondary tumours, gastrointestinal tract

## Abstract

Metastases to the colon are rare and may mimic other diseases such as inflammatory bowel disease. This differential diagnosis is often overlooked by endoscopists which leads to unnecessary diagnostic delay or inappropriate treatment. We present a case of a 54-year-old woman who presented with ascites. Malignant cells were demonstrated on cytologic examination of ascitic fluid, but no primary tumour or metastases were seen on computed tomography of the abdomen. On colonoscopy, an impassable stenosis was found in the transverse colon with scattered patches of oedematous colonic mucosa through to the mid-descending colon. Histological examination revealed scattered signet ring cells and the diagnosis of gastric signet ring cell adenocarcinoma was confirmed with subsequent gastroscopy. The correct and timely diagnosis of metastatic lesions to the colon requires a high index of suspicion and adequate mucosal sampling with multiple biopsies.

## Introduction

Secondary malignant tumours of the colon are rare and are estimated to account for 1%-3% of colonic tumours [1,2]. The most common primary tumours giving rise to colonic metastases include the prostate, ovary, breast, lung, and stomach [2,3]. The endoscopic appearance of metastatic lesions to the colon is nonspecific - they present as submucosal lesions, polypoid masses, dark spots, or ulcers, which can result in a significantly delayed diagnosis.

Signet ring cell adenocarcinoma is an aggressive malignancy characterized by mucin-producing cells, which most commonly arises from the stomach, but has also been reported from the breast, colon, and prostate [4]. It commonly metastasizes to the peritoneum and ovaries, while colonic metastases are rare and may mimic other diseases [5]. We present a case of a patient with colonic metastases of gastric signet ring cell adenocarcinoma.

## Case presentation

A 54-year-old woman with a past medical history of stress urinary incontinence presented with bloating and weight loss. In the month prior to evaluation, she noted increasing abdominal distension and anorexia resulting in a 3 kg weight loss. She reported intermittent regurgitation and heartburn. Her bowel habitat was regular and remained unchanged from her baseline. She had no family history of gastrointestinal malignancy or liver disease and no known exposure to tuberculosis.

The physical examination was notable for ascites without stigmata of chronic liver disease or signs of heart failure. Admission laboratory tests, including tumour markers carcinoembryonic antigen (CEA), carbohydrate antigen (CA) 19-9, CA 72-4, CA 15-3, and CA 125, were normal. Abdominal ultrasound confirmed the presence of ascites without liver masses. Gynaecological examination was unremarkable. Diagnostic paracentesis showed a low serum-ascites albumin gradient and cancer cells, consistent with adenocarcinoma. A computed tomography scan of the abdomen revealed ascites and mesenteric fat stranding suggestive of peritoneal carcinomatosis. No primary site of malignancy or distant metastases were identified.

**Figure 1:**
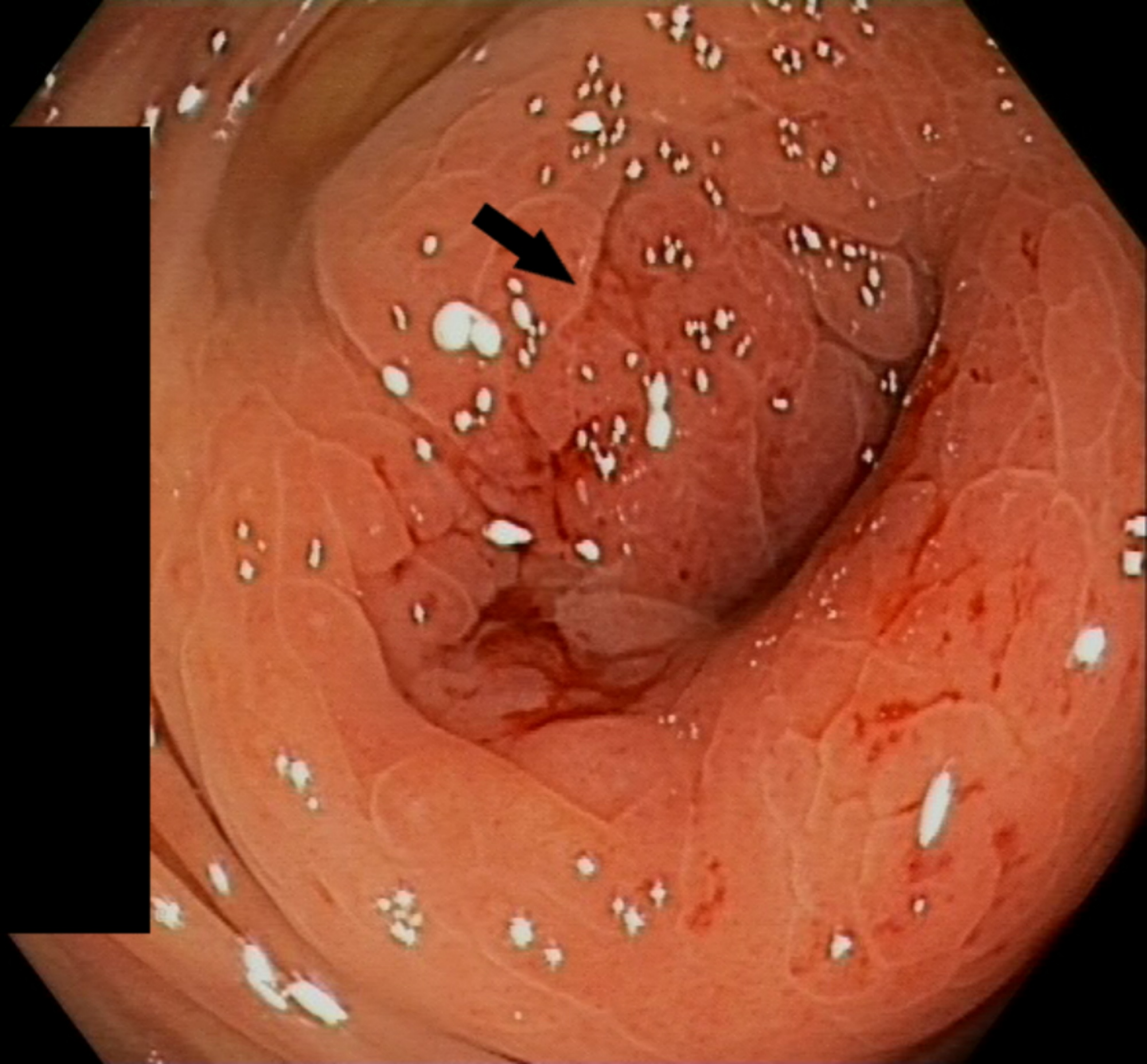
Endoscopic image of the stenosis in the transverse colon with metastatic deposits of gastric signet ring cell adenocarcinoma (arrow)

**Figure 2:**
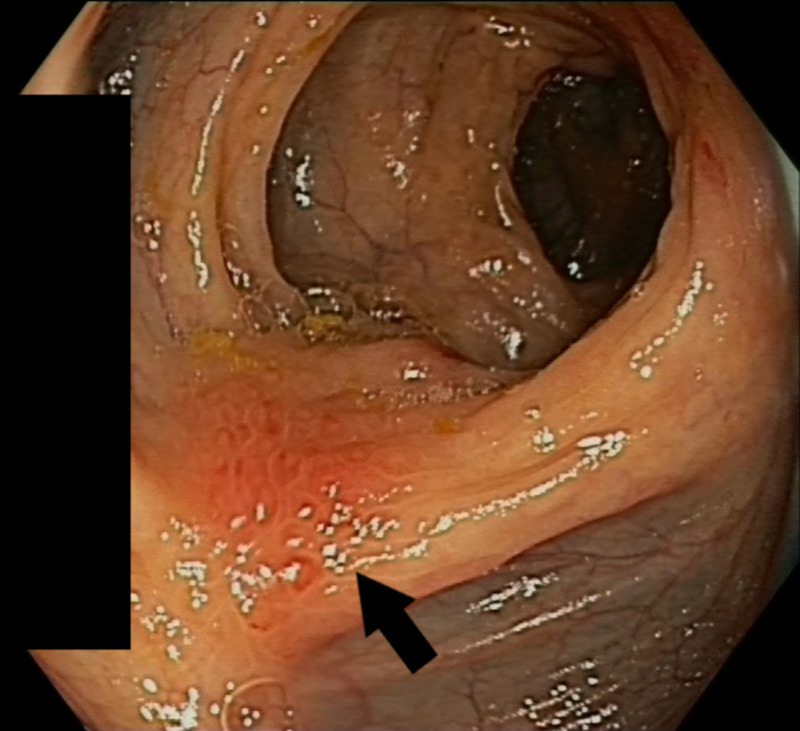
Metastatic deposit of gastric signet ring cell adenocarcinoma in the descending colon (arrow), macroscopically similar to the stenosis in the transverse colon

**Figure 3:**
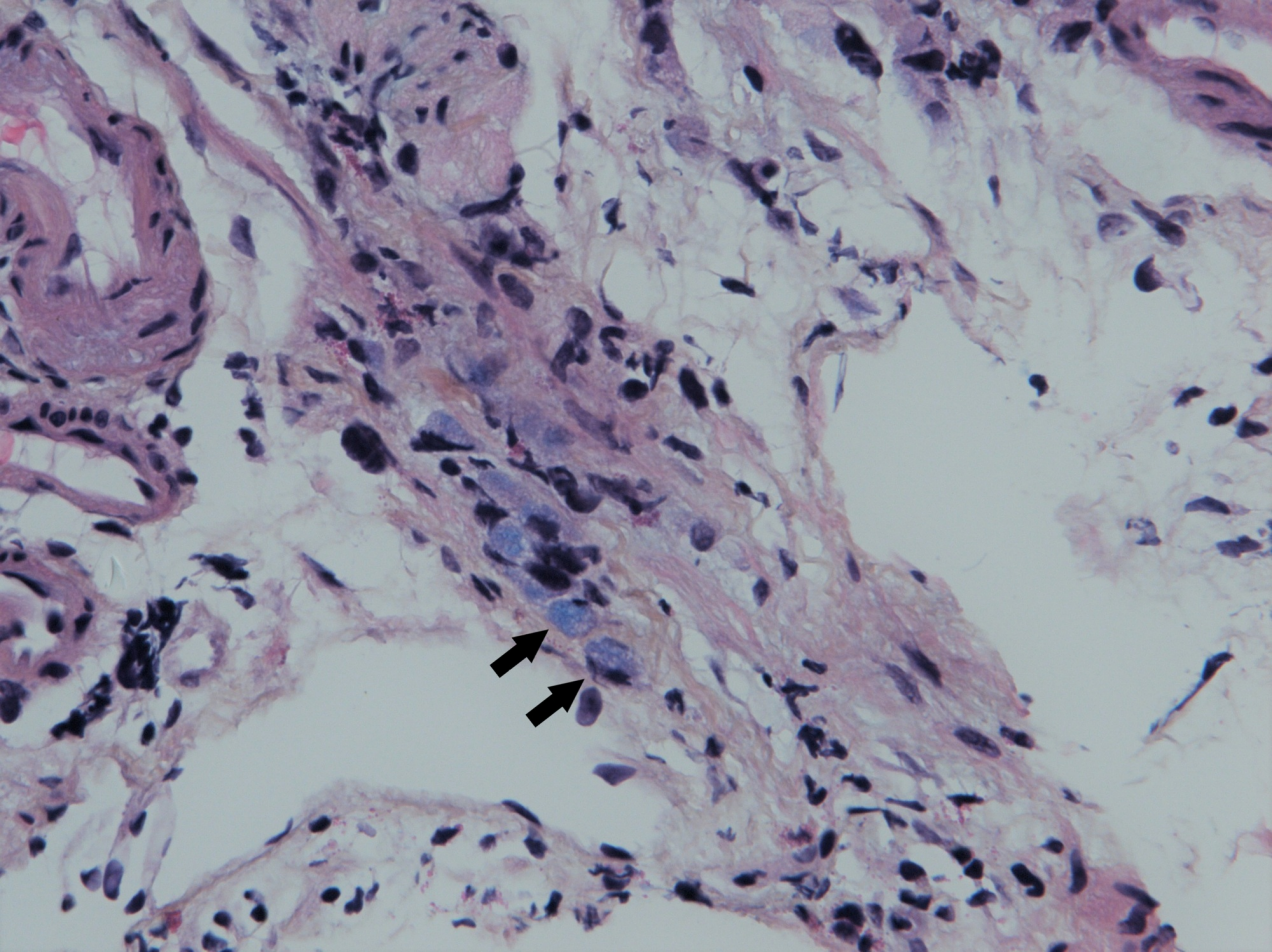
Microscopic image of the transverse colon; Kreyberg stain, 40 x. Signet ring cells are highlighted with arrows

A colonoscopy was performed because of the high clinical suspicion of gastrointestinal malignancy. In the proximal transverse colon, a stenosis that could not be passed was found (Figure 1). The mucosa was oedematous without ulceration. A few isolated patches of flat elevated mucosa with similar appearance were scattered distally to the mid-descending colon with normal intervening mucosa (Figure 2). Histopathologic examination showed superficial samples of the colonic mucosa with preserved architecture and a mild non-specific inflammatory infiltrate. In the few samples containing submucosa, scattered mucin-rich cells (arrows) were seen (Figure 3).

**Figure 4:**
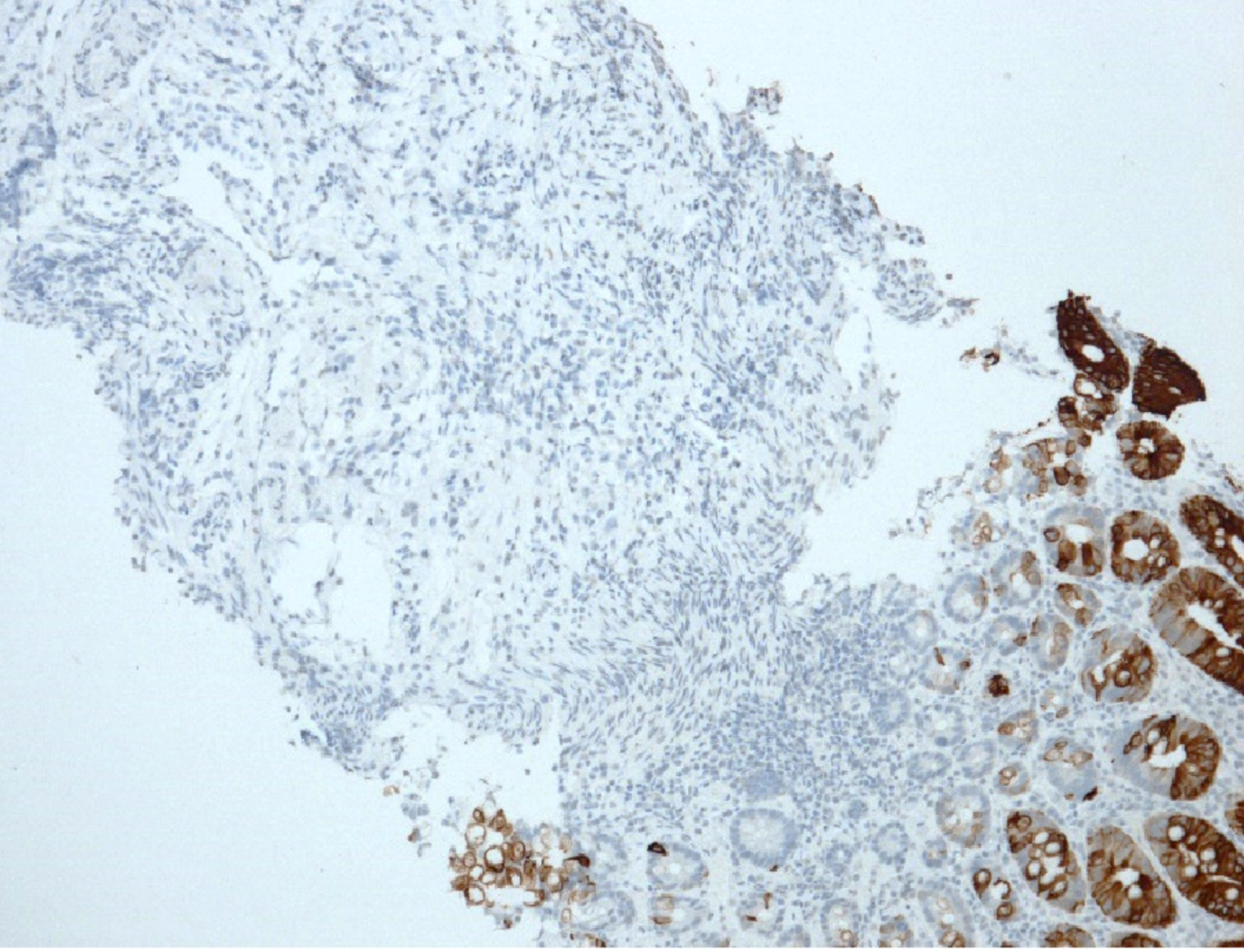
Immunohistochemical staining for special adenine-thymine-rich sequence-binding protein 2 (SATB2) of the colonic biopsy samples. The colonic epithelium is stained, while the tumour cells in the submucosa are not, confirming that the tumour originated outside the colon

The histopathology was consistent with metastatic signet ring cell carcinoma and subsequent gastroscopy confirmed the primary tumour. Negative staining of the colonic samples for special adenine-thymine-rich sequence-binding protein 2 (SATB2) supported the diagnosis of secondary deposits of gastric adenocarcinoma (Figure 4).

In the absence of obstructive symptoms, treatment of the colonic stenosis was not pursued in our patient. She was started on palliative chemotherapy and died nine months after diagnosis - without developing symptoms of colonic obstruction.

## Discussion

Colonic metastases of gastric signet ring cell adenocarcinoma are rare, with fewer than 100 cases reported in the literature [6-9]. Metastases to the colon are often misdiagnosed, even in the context of a known primary tumour, the differential diagnosis of metastatic deposits was suspected during endoscopy in only 48% of cases [3].

Metastases of gastric signet ring cell adenocarcinoma to the colon can present as granular and oedematous mucosa [6], polypoid lesions [7], mucosal ulceration [9], stenosis or a combination of these as observed in our patient. The findings are typically multifocal. The nonspecific endoscopic appearance and the sparse distribution of signet ring cells in the colonic submucosa can result in misdiagnosis or diagnostic delay. In an earlier study, the diagnosis was confirmed with endoscopic biopsies in a third of cases, half of the patients required a laparotomy for diagnosis, which was only confirmed on autopsy in the remainder of cases [6]. Inflammatory bowel disease is a common misdiagnosis for these lesions [6,9], especially in patients without other signs suggesting malignancy, such as peritoneal carcinomatosis in our case.

Gastric cancer is thought to spread to the colon either directly along the gastrocolic ligament to the transverse colon or through peritoneal carcinomatosis, although rare cases of presumed lymphogenic spread with involved lymph nodes and no peritoneal carcinomatosis have also been described [8]. The colonic involvement is usually multifocal with single metastatic deposits being the exception [8]. The prognosis of gastric signet ring cell carcinoma is worse than for other forms of gastric cancer with a median survival of 13 months [10]. The worse prognosis is mainly driven by the higher frequency of distant spread at diagnosis compared to other histologic subtypes, as the difference in survival was not significant when adjusted for disease stage and age at presentation.

## Conclusions

Our case highlights the importance of careful endoscopic evaluation and generous sampling for the correct diagnosis of metastases to the colon. Although the timely diagnosis of colonic metastases of gastric signet ring cell adenocarcinoma does not necessarily have therapeutic consequences in view of the poor prognosis, it may prevent unnecessary surgery or inappropriate treatment in case of suspected inflammatory bowel disease. Clinicians should retain a high index of suspicion for malignancy in seemingly inflammatory lesions with inconclusive histology.
